# Draft Genome Sequences of Two Drug-Resistant *Mycobacterium tuberculosis* Isolates from Myanmar

**DOI:** 10.1128/genomeA.00850-16

**Published:** 2016-10-27

**Authors:** Htin Lin Aung, Thanda Tun, Elizabeth Permina, Wint Wint Nyunt, Si Thu Aung, Kyi Kyi Thinn, John A. Crump, Gregory M. Cook

**Affiliations:** a Department of Microbiology and Immunology, Otago School of Medical Sciences, University of Otago, Dunedin, New Zealand; b Maurice Wilkins Centre for Molecular Biodiscovery, University of Auckland, Auckland, New Zealand; c National Health Laboratory, Ministry of Health and Sports, Yangon, Myanmar; d Otago Genomics and Bioinformatics Facility, University of Otago, Dunedin, New Zealand; e National TB Programme (NTP), Ministry of Health and Sports, Naypyidaw, Myanmar; f Department of Microbiology, University of Medicine 1, Yangon, Myanmar; g Centre for International Health, Dunedin School of Medicine, University of Otago, Dunedin, New Zealand

## Abstract

Multidrug-resistant tuberculosis (MDR-TB) and lately, extensively drug-resistant TB (XDR-TB) are increasing global health concerns. Here, we present the genome sequences of two MDR-TB isolates from Myanmar, one of 27 countries with a high MDR-TB burden, and describe a number of mutations consistent with these being XDR-TB isolates.

## GENOME ANNOUNCEMENT

Myanmar is 1 of 22 high-burden tuberculosis (TB) countries with a high prevalence of multidrug-resistant TB (MDR-TB) (resistant to rifampin and isoniazid) of 5% among new cases and 27% among re-treatment cases in 2013 (1, 2). Rapid detection of drug resistance is essential to effectively manage patients with drug-resistant TB. The National TB Reference Laboratory performs genotypic testing with the Hain GenoType MTBDR*plus* v1.0 (Hain Lifescience GmbH, Nehren, Germany) and phenotypic drug susceptibility testing (DST) of four first-line drugs, isoniazid, rifampin, ethambutol, and streptomycin. However, second-line drugs or pyrazinamide is currently not performed as part of the routine diagnosis of drug-resistant TB. Therefore, little is known about the prevalence of resistance to amikacin, pyrazinamide, levofloxacin, ethionamide, and cycloserine, the drugs used in the MDR regimen in Myanmar (3).

To examine drug resistance to second-line agents in Myanmar, we employed whole-genome sequencing (WGS) as the surrogate for phenotypic resistance and sequenced two MDR-TB isolates (M78 and M67) from Yangon, Myanmar. Both isolates are resistant to isoniazid, rifampin, ethambutol, and streptomycin by phenotypic DST. The genomic DNA of these two isolates was sequenced using paired-end 250-bp reads on an Illumina MiSeq (Illumina, Inc., Hayward, CA). A total of 1,070,097 and 1,367,219 paired-end reads from M78 and M67, respectively, were mapped to the *M. tuberculosis* H37Rv reference genome (accession no. AL123456.3) by BWA (4), yielding about 68× and 87× coverage. Single-nucleotide polymorphism (SNP) analysis was performed using GATK pipeline (5) to identify mutations associated with resistance. Final genome assembly was performed using a combination of tools, including Edena v3 (http://www.genomic.ch/edena.php) and FastaAlternateReferenceMaker (5). Ethical approval for this study was given by the Research and Ethical Committee of the University of Medicine 1, Yangon, Myanmar.

Consistent with phenotypic DST results, we identified mutations associated with resistance to rifampin (S450L in *rpoB*), isoniazid (S315T in *katG*), ethambutol (M306V in *embB*), and streptomycin (K43R in *rpsL*) in M78. Similarly, H445R in *rpoB*, S315T in *katG*, M306I and G406D in *embB*, and K43R in *rpsL* were identified in M67. Regarding second-line drug resistance, mutations associated with resistance to fluoroquinolones such as levofloxacin (A90V in *gyrA*) and aminoglycosides such as amikacin (G1484T in *rrs*) were identified in M78. Likewise, a G1484T *rrs* mutation was encountered in M67. Interestingly, heteroresistance to fluoroquinolones was identified at the *gyrA* gene in M67 with D94A and D94G present at 60% and 37% of total reads correspondingly. The *pncA* I133L and D12G mutations associated with resistance to pyrazinamide were identified in M78 and M67, respectively. Despite the lack of phenotypic DST results, identification of high-confidence mutations conferring resistance to both fluoroquinolones and aminoglycosides suggested that these strains are highly likely to be XDR isolates. This would also reduce the number of effective drugs to only two (ethionamide and cycloserine) in the standard MDR regimen. This underscores the need to introduce second-line DST in routine diagnosis in Myanmar as described previously (6) to enable construction of effective regimens for treatment of drug-resistant TB patients. Doing so will also allow estimation of the prevalence of extensively drug-resistant TB in the country despite the first reported case in 2007 (7).

### Accession number(s).

This whole-genome shotgun project has been deposited at DDBJ/EMBL/GenBank under the accession numbers MCQX00000000 (M67) and MCQY00000000 (M78). The versions described in this paper are the first versions.
